# General health of students of medical sciences and its relation to sleep quality, cell phone overuse, social networks and internet addiction

**DOI:** 10.1186/s13030-019-0150-7

**Published:** 2019-05-14

**Authors:** Rasool Kawyannejad, Maryam Mirzaei, Ali Valinejadi, Behzad Hemmatpour, Hasan Ali Karimpour, Javad AminiSaman, Ebrahim Ezzati, Siavash Vaziri, Mojgan Safaeepour, Saeed Mohammadi

**Affiliations:** 1 Department of Anesthesia, School of Allied Medical Sciences, Kermanshah University of Medical Sciences, Kermanshah, Iran; 2 Department of Rehabilitation and Sports Medicine, School of Allied Medical Sciences, Kermanshah University of Medical Sciences, Kermanshah, Iran; 3 Department of Health Information Technology, School of Allied Medical Sciences, Semnan University of Medical Sciences, Semnan, Iran; 4 Department of Anesthesia, School of Medicine, Kermanshah University of Medical Sciences, Kermanshah, Iran; 5 Department of Infectious Disease, School of Medicine, Kermanshah University of Medical Sciences, Kermanshah, Iran

**Keywords:** General health, Sleep quality, Internet addiction, Student

## Abstract

**Background:**

In recent years, the phenomena of access to the cell phone and addiction to the Internet have been developed among students due to their many applications and attractiveness. Therefore, the present study was conducted with the aim of evaluating general health status and also determining the predictive role of variables such as cell phone usage, sleep quality, internet addiction and social networks addiction in students.

**Methods:**

This cross-sectional study was conducted on 321 students of Kermanshah University of Medical Sciences in an analytical approach. Data collection tools were: Goldberg’s General Health Questionnaire, Pittburgh Sleep Quality Index, Young Internet Addiction Test, Social Network Addiction Questionnaire, and Cell Phone Overuse Scale. Data analysis was done using SPSS version 21 and general linear model.

**Results:**

Based on the results, the mean (SD) score of the general health was 21.27 (9.49). Variables of gender, sleep quality, and levels of cell phone usage were independent predictors of student’s health. Male students (β (95% CI) = − 0.28 (− 0.49 to − 0.01) and students with favorable sleep quality (β (95% CI) = − 0.22 (− 0.44 to − 0.02) had lower total health score than the reference category (female students and students with unfavorable sleep quality, respectively). In addition, students with cell phone overuse (β (95% CI) = 0.39 (0.08 to 0.69) had a higher general health score than the reference category (students with cell phone little use).

In general, this group of students had lower general health status (Low or high scores of general health indicate a higher and lower general health status for subjects, respectively).

**Conclusion:**

Variables of gender, sleep quality and cell phone use were the most important variables associating the general health of medical students.

## Background

General health as a substantial basis of the health system consists of overarching social activities and actions that are mainly based on the prevention strategy [1]. Since the health of the students guarantees the level of science and development of each community, attention to promoting the general health of students of medical sciences as human resources and responsible persons for the health of community and also identifying the factors affecting this variable seems necessary [2–4]. In Iran, numerous studies have been conducted on the general health of students, and the results of these studies can provide valuable information about educational planning to the authorities. According to Jahanbani et al., 11.8% of students did not have good general health [5]. According to a review and meta-analysis study that combined the results of 77 studies in 2016, the prevalence of mental disorders among Iranian universities based on the general health questionnaire was estimated to be 33% (95% CI = 0.29–0.37) [6]. Technology and its daily communication are among the most influential factors on health [3, 7]; On the other hand, in recent years, the phenomenon of access and addiction to the Internet and social networks has been developed among students due to its various applications and attractiveness [7]. Since educational environments such as universities are the places of Internet addiction development, by cell phone overuse and spending lots of time on the Internet students can be exposed to abnormal patterns of sleep, lifestyle changes and weak academic performance [8, 9].

The lifestyle changes of students can threaten the general health of students as a result of using Internet and cell phone, and may cause a loss in active human resources [6, 8]. Surveys on general health of students have shown that variables such as gender, cell phone overuse, addiction to social networks, sleep quality, etc. affected the general health. In a research conducted in 2009 on students of Zahedan University of Medical Sciences, reported the association of the general health of participants with the level of Internet addiction; in the other word, general health and its dimensions were more threatened among the Internet addicts compared to normal users [10]. In the study of Bahri et al. [11] the general health score has shown a significant and negative statistical relationship with addiction to the Internet. Other studies have reported a significant positive correlation between general health dimensions and cell phone overuse in students; besides, the disturbance of students’ sleeping patterns due to cell phone overuse, as well as psychological demands and pressures of academic environments were common among students [12–14].

Therefore, it seems that sleep quality, Internet addiction, and cell phone overuse and their relationship with general health in the students need to be examined.

To date, In Iran, few studies have investigated the relationship between general health and variables such as Internet and cell phone addiction, but in interpreting the results cultural differences and other factors such as different usage rate of Internet and cell phone should have been considered for the students of different universities [11, 12, 15].

Until now, however, general health and the factors associating the general health among medical students in Iran have received minimal attention and little is known about the factors associating the general health in this group. As mentioned above, according to the serious concerns about the association of cell phone overuse, Internet and social networks on health, the present study was conducted to determine better understand the students’ general health status and its relationship with cell phone overuse, Internet and social networks, as well as the sleep quality (as one of the key indicators of general health) on the students of Kermanshah University of Medical Sciences. The results of this study can be useful to identify the factors associating general health and preventative actions to eliminate these factors in medical students.

## Methods

### Participants

After obtaining ethical permission from Ethics Committee of Kermanshah University of Medical Sciences in 2016, this cross-sectional study was conducted on 321 students of medical sciences in Kermanshah University of Medical Sciences through random cluster sampling. In the first semester of 2016 in Kermanshah University of Medical Sciences, a sample size of 350 out of 5000 students was randomly selected using Morgan table with a 95% confidence interval and 5% error. They were selected from all levels (bachelor, master, G.P.) and disciplines (paramedical, medical, nursing, dentistry, health, and pharmacy) (out of 350 samples, 29 did not return the questionnaire). The inclusion criteria included being a second year student or above, using smartphone and social networks applications, and being an indigenous student of Kermanshah and not living in dormitories. Before the commencement of the study, participants were informed of the objectives and importance of the survey. All the subjects were also assured that the data were confidential and they were collected just for the current research.

### Instruments

In the present study, the main variables included general health, sleep quality, cell phone overuse, social networks addiction, age, and gender. The data collection tools were General Health Questionnaire (GHQ28), Pittsburg Sleep Quality Index, Social Network Addiction Questionnaire, and Cell Phone Overuse Scale.

***General Health Questionnaire (GHQ-28)*** is a psychometric screening tool to examine the mental health in the recent month. It comprises 28 questions, each with a four-point Likert scale for responses. It was developed by Goldberg and Hillier. GHQ-28 is divided into four components ((1) somatic symptoms, (2) social dysfunction, (3) anxiety and insomnia and (4) severe depression); and each subscale comprises 7 items. The scoring range is from 0 to 84. After scoring, the total score of general health is calculated from the total score of the questions. Based on the study of Norbala et al. [15] the cut-off point for the general health score was determined 23. Less and more scores below this cut-off point, respectively, indicated a higher and lower general health status for subjects. Validity and reliability of the original questionnaire as well as the Persian version have been confirmed in various studies [15, 16].

***Pittburgh Sleep Quality Index*** investigates the attitude towards sleep quality in the last 4 weeks. It has seven component scores including subjective sleep quality, sleep latency, sleep duration, habitual sleep efficiency, sleep disturbances, use of sleeping medication, and daytime dysfunction. Each item is scored on a 0–3 Likert scale. The total score is calculated, ranging from 0 to 21. Higher score denotes a lower quality sleep. The total score above 5 indicates a significant sleep disorder. Validity and reliability of this questionnaire in Iran were confirmed by Cronbach’s alpha (0.77) in the study of Moghaddam et al. [17].

***Cell Phone Overuse Scale*** is designed based on the psychological index of the Diagnostic and Statistical Manual of Mental Disorders. It has 23 items and scoring is based on a 6-point Likert scale. The score above 75 shows overuse, the score between 25 and 75 demonstrates normal use, and the score below 25 shows low usage [18]. Validity and reliability of this questionnaire in Iran were confirmed by Cronbach’s alpha (0.90) in the study of Golmohammadian et al. [12].

***Young Internet Addiction Test (YIAT)*** is a reliable and valid questionnaire including 20 items being assessed through a five-point Likert scale [7]. The minimum score is 20 and the maximum is 100. Based on the participants’ scores, three groups were classified as following: no Internet addiction (score 20–49), exposed to the Internet addiction (50–79), and Internet addiction (80–100) [7].

***Social Network Addiction Questionnaire*** is a researcher-made questionnaire, which comprises 15 questions about knowing social networks (Facebook, Orkut, MySpace, Viber, Line, Tango, WeChat, Telegram, Whatsapp, Instagram, Twitter, LinkedIn, YouTube, Status, etc.) and using them. The scoring is based on a five-point Likert scale (from 5 = always to 1 = rarely). For developing this researcher-made questionnaire various authentic scientific texts, similar studies, and experts’ opinions in this field were used. Its validity was confirmed by five faculty members and its reliability was confirmed by Cronbach’s alpha (0.83). Assessing and scoring were as following: total score from 1 to 25 assumed as a normal user; the scores from 26 to 49 suggested that the user was exposed to get addicted to social networks and it was better to prevent it; and scores from 50 to 75 considered that the user was addicted to social networks and the user should think about the treatment as soon as possible [19].

In the present study, the reliability of all the used questionnaires was confirmed by the alpha coefficient of 0.7.

### Statistical tests

The descriptive statistics of mean (standard deviation (SD)) and frequency (percentage) were used to describe the variables.

The probability of normal distributions of data was confirmed by Kolmogorov-Smirnov test for quantitative variables (*P* > 0.05). Pearson correlation analysis was used to assess the association between the quality of sleep and general health subscale.

General linear model (GLM) was used to determine the relationship between the variables including gender, age, internet addiction cell phone overuse, sleep quality and general health’s total score, as well as to determine the proportion of each variable in the general health score. Qualitative variables were introduced into the analysis as dummy variables; first, each unadjusted variable was entered into the GLM, then based on single-variable tests the variables with *P* < 0.05 by the adjustment of other variables entered the GLM. Data analysis was performed by SPSS version 21 (SPSS Inc. Chicago, II, USA); and the significance level was determined less than 0.05.

## Results

The mean (SD) age of the students participating in the survey was 22.03(1.81) years; and 39.9% of them were male students. The amount of real sleep in the participants was 6.58 (1.05) hours; and the ranging score was from 0 to 21. Besides, 53.9% of students had poor sleep quality. The duration of daily cell phone use was 3.32 (1.42) hours; and the duration of daily activity in social networks was 3.87 (2.87) hours, as 31 subjects (9.7%) had social networks addiction (Table 1). The mean (SD) total score of general health in students was 21.27 (9.04), while the minimum score was 5 and the maximum was 66. Total score of general health by the studied variables is shown in Table 2. The highest scores of general health were seen in students who had cell phone overuse and addiction to the Internet and social networks, as well as in the female students.

**Table 1:** Frequency distribution and percentage of students according to the variables (*n* = 321)

Variable	Categories	Frequency (percentage)
Gender	Male	128 (39.9)
	Female	193 (60.1)
Social networks addiction	No social networks addiction	116 (36.1)
	Exposed to the social networks addiction	174 (54.2)
	Social networks addiction	31 (9.7)
Internet addiction levels	No Internet addiction	215 (67)
	Exposed to the Internet addiction	83 (25.9)
	Internet addiction	23 (7.2)
Cell phone overuse	Overuse	29 (9)
	Normal use	241 (75.4)
	Little use	50 (15.6)
Sleep quality	Favorable	167 (52)
	Unfavorable	154 (48)
Age (years)^a^		22.03 (1.82)

^a^Mean (SD)

**Table 2:** The mean (SD) total score of general health in the students according to the variables (*n* = 321)

Variables	Categories	Mean (SD)
Gender	Male	19.93 (8.42)
	Female	22.16 (9.38)
Social networks addiction	No social networks addiction	19.95 (9.12)
	Exposed to the social networks addiction	21.89 (8.88)
	social networks addiction	22.71 (9.26)
Internet addiction levels	No Internet addiction	21.17 (9.39)
	Exposed to the Internet addiction	20.81 (7.96)
	Internet addiction	23.91 (9.26)
Cell phone overuse	Little use	20.51 (8.97)
	Normal use	22.06 (8.33)
	Overuse	24.50 (9.19)
Sleep quality	Favorable	20.07 (8.66)
	Unfavorable	22.17 (9.22)

Furthermore, there were significant moderate correlations between quality of sleep and the total score of general health (r = 0.506, *p* < 001) and its four components (somatic symptoms (*r* = 0.381) social dysfunction (*r* = 0.301) anxiety and insomnia (*r* = 0.325) and severe depression (0.341); all *p* value < 0.001).

The relationship between the variables and the general health of students is shown in Table 3.

**Table 3:** Comparison of general health’s total score with variables based on univariate and multivariate general linear model (*n* = 321) (depended variable: General health)

Variables	β (95%CI)	Unadjusted *P*-Value	β (95%CI)	Adjusted *P*-Value
Gender	Female (reference)			
	Male −0.25 (− 0.48 to − 0.03)	0.031	−0.28 (−0.49 to −0.01)	0.019
Social networks addiction	Social networks addiction (reference)			
	No Internet addiction 0.31 (− 0.09 to 0.71)	0.131	–	–
	Exposed to the social networks addiction 0.10 (− 0.47 to 0.29)	0.644	–	–
Internet addiction levels	Internet addiction (reference)			
	No Internet addiction 0.32 (− 0.13 to 0.73)	0.913	–	–
	Exposed to the Internet addiction 0.35 (− 0.12 to 0.81)	0.254	–	–
Cell phone overuse	Little use (reference)			
	Normal use 0.27 (− 0.19 to 0.73)	0.245	0.23 (− 0.71 to 0.25)	0.354
	Overuse 0.44 (0.13 to 0.75)	0.044	0.39 (0.08 to 0.69)	0.014
Sleep quality	Unfavorable (reference)			
	Favorable − 0.24 (− 0.46 to- 0.02)	0.039	−0.22 (−0.44 to − 0.02)	0.041
Age (years)	0.06 (− 0.17 to 0.06)	0.230	–	–

Based on the univariate analysis, there was a significant relationship between the mean total score of general health, gender, cell phone overuse, and sleep quality levels (*p* < 0.05). Then, based on the multivariate analysis and by adjusting other variables, the gender, cell phone overuse and sleep quality found a significant relationship with general health score; these variables predicted 20% of general health variance in medical students (Table 3). Overall, male students (β (95%CI) = − 0.28 (− 0.49 to − 0.01) and students with favorable sleep quality (β (95%CI) = − 0.22 (− 0.44 to − 0.02) had lower general health scores than the reference category (female students and students with unfavorable sleep quality, respectively).

Moreover, students with over use (β (95%CI) =0.39 (0.08 to 0.69) of cell phone had higher general health scores. In the other words, students with over use of cell phone are less healthy than the reference category (students with cell phone little use).

## Discussion

In the present study the mean score of general health was at a favorable level. There was a significant relationship between sleep quality and general health. In addition to the level of sleep quality, the gender and cell phone overuse were also other associate factors of general health in students.

The mean total score of general health in the students was 21.27 (9.04) (the scores ≤23 in health were considered as favorable scores). According to other national studies, the mean (SD) general health in students of Gonabad University of Medical Sciences, Rasht Midwifery School, and Urmia University of Medical Sciences were respectively 25.84 (11.97), 22.54 (11.22), 23.42 (22.09) [11, 20, 21].

In the current study, one of the associate factors was the gender variable. It is seen that general health’s total score in male students was less than in the reference category (female students); and the general health in male students was more favorable than in female students (*P* < 0.05). Various studies have reported significant relationship between the general health’s total score and the student’s gender; these results were consistent with results of the current study [22–24]. On the contrary, this significant relationship between the general health’s total score and the student’s gender was not seen in the studies of Hashemi et al., Mosalanejad et al., and Sotodeh et al. [5, 25, 26]. Lower quality of general health in female students can be attributed to issues such as the discrimination and cultural differences existing in the society of Iran between men and women (e.g. constraints, lack of sports facilities, and unreasonable prejudice towards women), which negatively affects women’s general health [23, 24].

In the present study, based on the variable of cell phone, the highest mean scores for general health were seen in students with overuse of cell phone. This indicates that students who had over use of cell phone had lower general health status than the reference category (students with cell phone little use). These findings are consistent with the results of similar studies in this field [10, 12, 27, 28].

The Yaseminejad et al. indicated that general health score was significantly associated with cell-phone over use in students [12]. Similar results can be attributed to the fact that students who have overuse and unnecessary use of cell phone may neglect many of their daily activities. Besides, cell phone overuse results in improper behavior patterns, such as staying awake at night and exchanging messages; this results in disturbances in sleep patterns and, consequently, weakening their general health [29]. In sum, these findings support the negative impact of cell phone overuse on students’ physical and psychological health [30].

In this study, GHQ total score in subjects with poor sleep quality was higher than those with good sleep quality. So that the group with favorable sleep level had higher level of general health than the reference category (students who had unfavorable sleep level). Previous studies have shown that the variable of sleep quality has played an important role in the general health of students, and the increase of favorable sleep level could improve the general health, and as a result it could improve the quality of life and academic performance of students. Mohammadi et al., in their study in 2012, found a positive and significant relationship between the sleep quality and general health [31]. Likewise Chegini et al. in their research stated that the scores of sleep quality had strong and significant correlation with the general health’s total score [32]. According to the results of the present study that indicate the positive relationship between general health and cell phone overuse, there is a presumption that cell phone addiction affects the quality of medical students’ sleep, and consequently abnormal sleeping pattern in students negatively affects their general health [29, 33].

Findings of the present study showed no significant relationship between age, levels of Internet and social networks addiction and the general health score. These findings are consistent with other studies in this field, for instance, Heravi et al.’s study on Tehran’s nursing students indicated that there was no significant correlation between age and general health score [23].

On the other hand, regarding the lack of significant relationship between the levels of Internet and social networks addiction and the general health’s total score, the results of the current study were not consistent with the results of some similar studies in this regard [10, 11, 34]. This inconsistency of results can be due to some issues such as the difference in nature of populations, the difference in the evaluation methods and reporting the studied variables, cultural and social differences, and universities’ welfare facilities.

As the main strengths of the study we can mention the appropriate sample size and the use of valid and reliable questionnaires. The current study has some limitations. First, due to the cross-sectional nature of the present study, it is not possible to deduce the causal relationship. Moreover, since the study subjects were limited to medical sciences students and they are not represent the general population of young adults, results of the present study cannot be generalized to the general population of young adults. Along with the consideration of more variables in the future, conducting prospective studies is recommended to confirm the causal relationship of the variables.

## Conclusion

The present study reviewed the general health and some of its predictive factors in students of Kermanshah University of Medical Sciences. The mean score of general health was moderate. Variables such as gender, sleep quality, and cell phone overuse were associated of general health. Since general health affects many aspects of students’ life, including their academic achievement, identifying health-related factors can be fruitful in planning for enhancing factors of general health in medical students.

## Abbreviations

GHQ-28: General Health Questionnaire
GLM: General linear model
SD: Standard deviation
YIAT: Young Internet Addiction Test
